# Association Between Atherogenic Index of Plasma and Patients With Acute Ischemic Stroke Receiving Intravenous Thrombolysis: A Retrospective Cohort, Multi-Center Study

**DOI:** 10.31083/RN40923

**Published:** 2025-11-30

**Authors:** Rongrong Shao, Zhengyang Wang

**Affiliations:** ^1^Department of Neurology, Shanghai Fifth People’s Hospital Affiliated to Fudan University, 200240 Shanghai, China; ^2^Department of Neurology, Taizhou Clinical Medical School of Nanjing Medical University, Jiangsu Taizhou People’s Hospital, 225300 Taizhou, Jiangsu, China

**Keywords:** atherogenic index of plasma, acute ischemic stroke, intravenous thrombolysis, risk factor, índice aterógeno del plasma, accidente cerebrovascular isquémico agudo, trombólisis intravenosa, factor de riesgo

## Abstract

**Objectives:**

There are inherent risks associated with intravenous thrombolysis (IVT) therapy in patients with acute ischemic stroke (AIS). The atherogenic index of plasma (AIP), defined as log (triglyceride [TG]/high-density lipoprotein cholesterol [HDL-C]), has recently been associated with the prognosis. We aimed to gauge AIP prognostic value in AIS patients receiving IVT.

**Methods:**

We retrospectively collected data from 183 AIS patients who underwent IVT. We grouped modified Rankin Scale scores of 0–2 and 3–6 as good and poor outcomes at 1 year, respectively. Multivariate logistic regression, receiver operating characteristic (ROC) curve and restricted cubic spline (RCS) analyses were used to investigate the underlying link between the AIP and 1-year functional outcomes.

**Results:**

In this study, 67 patients (36.6%) exhibited poor 1-year outcomes. An optimal AIP cut-off of 0.188 was used to divide the patients into low and high AIP levels. Our results showed that continuous AIP (odds ratio [OR] = 25.10, 95% confidence interval [CI]: 4.86–129.68, *p* < 0.001) was associated with poor 1-year outcome; when AIP was as a categorical variable, OR (95% CI) for the prognosis in the high AIP group was 27.86 (9.33–83.25) compared with the low AIP group. ROC analyses revealed that the area under the ROC curve for the AIP was 0.694 (0.603–0.785), with a sensitivity of 87.1% and a specificity of 61.2%. In the fully adjusted RCS, we found a positive but non-linear trend between the AIP and prognosis.

**Conclusions:**

High AIP may offer potential value as a novel target for predicting 1-year outcomes in patients receiving IVT.

## Abstract (es)

**Objetivos:**

Existen riesgos inherentes asociados al tratamiento con trombólisis intravenosa (IVT, *intravenous thrombolysis*) en pacientes con accidente cerebrovascular isquémico agudo (AIS, *acute ischemic stroke*). El índice aterógeno del plasma (AIP, *atherogenic index of plasma*), definido como log (triglicéridos [TG]/colesterol de lipoproteínas de alta densidad [HDLC]), se ha asociado recientemente con este pronóstico. Nuestro objetivo era evaluar el valor pronóstico del AIP en pacientes con AIS que reciben IVT.

**Métodos:**

Recopilamos retrospectivamente datos de 183 pacientes con AIS que se sometieron a IVT. Agrupamos las puntuaciones de 0 a 2 y de 3 a 6 en la escala de Rankin modificada como buenos y malos resultados al año, respectivamente. Se utilizaron la regresión logística multivariable, la curva característica operativa del receptor (ROC, *receiver operating characteristic*) y análisis de splines cúbicos restringidos (RCS, *restricted cubic spline*) para investigar la relación subyacente entre el AIP y los resultados funcionales al año.

**Resultados:**

En este estudio, 67 pacientes (36,6%) mostraron resultados deficientes al año. Se utilizó un valor de corte óptimo del AIP de 0,188 para dividir a los pacientes en niveles bajos y altos de AIP. Nuestros resultados indicaron que el AIP continuo (*odds ratio* [OR] = 25,10, intervalo de confianza [IC] del 95%: 4,86–129,68, *p* < 0,001) se asociaba con un mal resultado al año. Cuando el AIP se consideraba una variable categórica, la OR (IC del 95%) para el pronóstico en el grupo con AIP alto era de 27,86 (9,33–83,25) en comparación con el grupo con AIP bajo. Los análisis de ROC revelaron que el área bajo la curva ROC para el AIP fue de 0,694 (0,603–0,785), con una sensibilidad del 87,1% y una especificidad del 61,2%. En el RCS totalmente ajustado, encontramos una tendencia positiva pero no lineal entre el AIP y el pronóstico.

**Conclusiones:**

Un AIP elevado puede ofrecer un valor potencial como nuevo objetivo para predecir los resultados a un año en pacientes que se someten a IVT.

## 1. Introduction

Acute ischemic stroke (AIS) is a debilitating and devastating disease, with a high global burden that is continuously increasing [1]. Although intravenous thrombolysis (IVT) with recombinant tissue-type plasminogen activator (rt-PA) has continued to occupy a pivotal position in the treatment of AIS, there are inherent risks associated with the process of IVT [2]. It is crucial to develop simple, non-invasive, and affordable biomarkers that could help assess prognosis and guide decision-making for eligible patients.

Atherosclerosis is a progressive disorder of arterial vessels. It is marked by the accumulation of lipids in the inner layer of the artery wall, increasing the incidence of AIS and cardiovascular disease (CVD) [3, 4]. Dyslipidemia is a key contributor to atherosclerosis, and is characterized by abnormal triglyceride (TG), total cholesterol (TC), high-density lipoprotein cholesterol (HDL-C), and low-density lipoprotein cholesterol (LDL-C) levels [5, 6]. These findings have spurred further interest in the prognostic relevance of lipid profiles in AIS. The atherogenic index of plasma (AIP) is defined as log (TG/HDL-C) and is negatively correlated with LDL-C levels [7, 8]. Therefore, the AIP serves as a metric to assess the severity of dyslipidemia in patients. Notably, several studies have observed an association between AIP and AIS prognosis [9, 10]. High AIP was correlated with the 3-month clinical outcomes in patients with AIS. However, data regarding the long-term prognosis of patients undergoing IVT are scarce. Therefore, we aimed to further explore the ability of the AIP to predict 1-year functional outcomes in a population of AIS patients receiving IVT by building on previous research [11].

## 2. Materials and Methods

### 2.1 Study Design 

To enhance data consistency and minimize loss to follow-up, 236 patients who underwent IVT and were admitted to Shanghai Fifth People’s Hospital (129 cases) and Taizhou People’s Hospital (107 cases) between January and December 2023 were enrolled between January and December 2023. Furthermore, all patients received standard statin therapy according to the guidelines [12]. Standardized telephone surveys were used to collect follow-up information.

The inclusion criteria for patients: (1) Aged ≥18 years. (2) Pretreatment modified Rankin Scale (mRS) score of 0–2. (3) Diagnosis of AIS was confirmed by head magnetic resonance imaging (MRI). The exclusion criteria: (1) Receiving bridging therapy; (2) Having hematologic diseases, active bleeding, severe heart, kidney, or liver failure, intracranial tumor; (3) being readmitted during the follow-up period; and (4) Incomplete or poor imaging/laboratory/follow-up information.

### 2.2 Data Collection

We collected demographic characteristics from the hospital records, including age, sex, body mass index (BMI), blood pressure, current smoking and drinking status, and medical history (including stroke or transient ischemic attack, coronary heart disease, atrial fibrillation, hypertension, and diabetes mellitus type 2). Neurological function was assessed using the NIH Stroke Scale (NIHSS) scores [13]. The Trial of ORG 10172 in Acute Stroke Treatment (TOAST) criteria were applied to categorize stroke subtypes [14].

### 2.3 Measurement of AIP

We gathered laboratory data, such as white blood cell (WBC), red blood cells (RBC), platelets (PLT), fasting plasma glucose (FPG), glycated haemoglobin A1c (HbA1c), TC, TG, HDL-C, and LDL-C. The AIP parameter was calculated as log(TG/HDL-C) [7].

### 2.4 Primary Outcomes

This study assessed the patients’ neurological function at 1 year. We classified mRS scores of 0–2 and 3–6 as indicating good and poor outcomes, respectively [15].

### 2.5 Statistical Analyses

All statistical analyses were performed using R software (version 4.4.1; R Foundation for Statistical Computing, Vienna, Austria). For categorical variables, the Chi-square test was applied. As for non-normally distributed continuous and ordinal variables, the Kruskal-Wallis test was employed. Multivariate logistic regression models were performed to explore the associations between continuous and categorical AIP and the 1-year functional outcomes. The best AIP cut-off value of 0.188 was determined corresponding the maximum Youden index (sensitivity – [1–specificity]) by the receiver operating characteristic (ROC) curve, with AIP divided into low and high levels. The crude model was a univariable analysis. Model 2 was adjusted for age, sex, BMI. In Model 3, we further adjusted for diastolic blood pressure (DBP), admission NIHSS, WBC, RBC, PLT, FPG, HbA1c, TC and LDL-C. In addition, we used ROC models to assess the predictive abilities of AIP and related lipid profiles. A fully adjusted restricted cubic spline (RCS) was applied to assess the associations of the continuous AIP with 1-year functional outcomes. Statistical significance was set at *p* < 0.05.

## 3. Results 

**Fig. 1.**
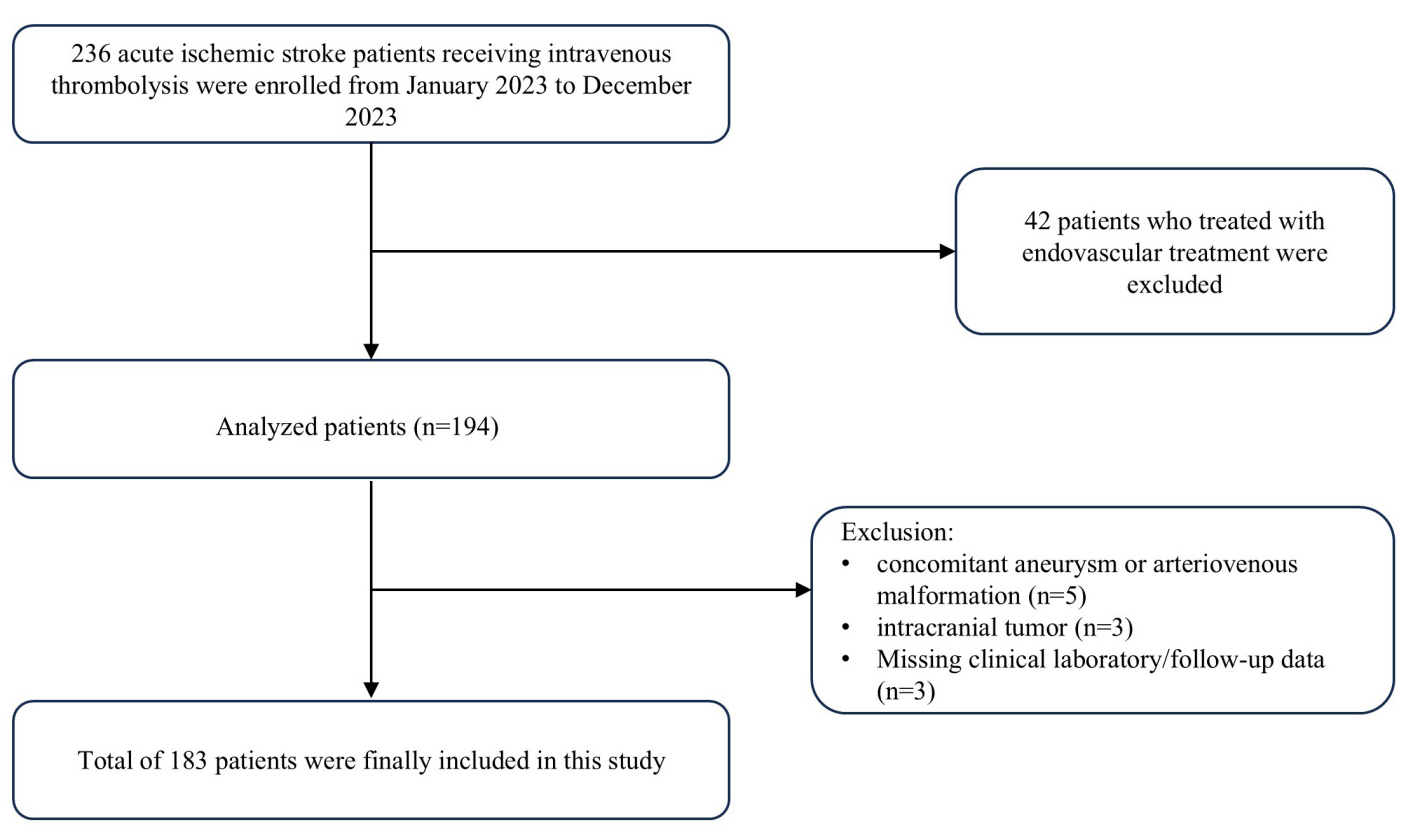
**Flowchart of the current study**.

After excluding a total of 53 patients (42 who received bridging therapy; 5 with concomitant aneurysm and/or arteriovenous malformation; 3 with intracranial tumor; 3 without complete data), 183 patients were finally selected. The flowchart is presented in Fig. 1.

The characteristics of subjects are presented in Table 1. The included participants had a mean age of 67.16 years, and 65.03% were men. In total, 67 patients (36.6%) had poor outcomes, whereas 73.4% had good outcomes. The AIP was significantly higher in the poor outcome group (0.15 ± 0.34) than in the good outcome group (–0.03 ± 0.22) (*p* < 0.001). Compared with the good outcome group, the poor outcome group was more likely to be men and had significantly higher diastolic blood pressure (86.00 [79.00, 97.50] vs. 81.00 [73.50, 91.25], *p* = 0.007), admission NIHSS (5.00 [3.00, 9.00] vs. 4.00 [2.00, 7.00], *p* = 0.030), WBC (7.44 [5.66, 9.75] vs. 6.64 [5.44, 8.05], *p* = 0.026), RBC count (4.64 [4.22, 4.95] vs. 4.33 [3.98, 4.82], *p* = 0.017), FPG (6.20 [5.46, 8.00] vs. 5.61 [4.93, 6.88], *p* = 0.015), HbA1c (6.00 [5.60, 7.40] vs. 5.90 [5.50, 6.50], *p* = 0.046), TC (4.60 [3.90, 5.29] vs. 4.20 [3.27, 5.08], *p* = 0.027), TG (1.49 [1.02, 2.15] vs. 1.10 [0.80, 1.40], *p* < 0.001), LDL-C (2.91 [2.34, 3.38] vs. 2.61 [1.89, 3.29], *p* = 0.039), and lower HDL-C levels (0.97 [0.86, 1.25] vs. 1.21 [1.01, 1.36], *p* < 0.001).

**Table 1.** **Baseline characteristics of included patients with acute ischemic stroke receiving intravenous thrombolysis according to 1-year functional outcomes**.

		Total	Good outcome	Poor outcome	*p *value
			(n = 116)	(n = 67)	
Demographics					
	Age, years	67.16 ± 12.24	67.31 ± 11.75	66.90 ± 13.15	0.432
	Male, n (%)	119 (65.03)	67 (56.30)	52 (43.70)	0.007
	Body Mass Index, kg/m^2^	23.31 (22.20, 25.60)	23.31 (22.20, 25.56)	23.26 (22.20, 25.54)	0.832
Clinical assessment					
	Systolic blood pressure, mmHg	150.00 (136.00, 163.00)	149.00 (136.00, 162.25)	150.00 (136.00, 163.00)	0.808
	Diastolic blood pressure, mmHg	82.00 (75.00, 94.00)	81.00 (73.50, 91.25)	86.00 (79.00, 97.50)	0.007
	Admission NIHSS	4.00 (3.00, 8.00)	4.00 (2.00, 7.00)	5.00 (3.00, 9.00)	0.030
	Time to admission, hours	3.00 (2.00, 4.00)	3.00 (2.00, 4.00)	3.00 (2.00, 4.50)	0.910
Medical history, n (%)					
	Smoking	57 (31.15)	32 (56.14)	25 (43.86)	0.171
	Drinking	37 (20.22)	24 (64.86)	13 (35.14)	0.835
	Hypertension	121 (66.12)	78 (64.46)	43 (35.54)	0.673
	Diabetes mellitus type 2	49 (26.78)	28 (57.14)	21 (42.86)	0.289
	Coronary heart disease	11 (6.01)	6 (54.55)	5 (45.45)	0.760
	Atrial fibrillation	28 (15.30)	19 (67.86)	9 (32.14)	0.594
	Stroke or Transient ischemic attack	19 (10.38)	10 (8.62)	9 (13.43)	0.304
Stroke subtype, n (%)					0.444
	Small-vessel	89 (48.63)	61 (68.54)	28 (31.46)	
	Large artery atherosclerosis	49 (26.78)	27 (55.10)	22 (44.90)	
	Cardioembolic	23 (12.57)	15 (65.22)	8 (34.78)	
	Undetermined or others	22 (12.02)	13 (59.09)	9 (40.91)	
Laboratory data					
	White blood cell, 10^9^/L	6.82 (5.50, 8.66)	6.64 (5.44, 8.05)	7.44 (5.66, 9.75)	0.026
	Red blood cell, 10^9^/L	4.40 (4.09, 4.85)	4.33 (3.98, 4.82)	4.64 (4.22, 4.95)	0.017
	Platelet, 10^9^/L	192.26 ± 59.06	187.17 ± 63.40	201.09 ± 49.89	0.125
	Fasting plasma glucose, mmol/L	5.90 (4.99, 7.38)	5.61 (4.93, 6.88)	6.20 (5.46, 8.00)	0.015
	HbA1c, (%)	5.90 (5.50, 6.70)	5.90 (5.50, 6.50)	6.00 (5.60, 7.40)	0.046
	TC, mmol/L	4.40 (3.60, 5.17)	4.20 (3.27, 5.08)	4.60 (3.90, 5.29)	0.027
	TG, mmol/L	1.15 (0.87, 1.68)	1.10 (0.80, 1.40)	1.49 (1.02, 2.15)	<0.001
	HDL-C, mmol/L	1.13 (0.93, 1.34)	1.21 (1.01, 1.36)	0.97 (0.86, 1.25)	<0.001
	LDL-C, mmol/L	2.78 (2.05, 3.35)	2.61 (1.89, 3.29)	2.91 (2.34, 3.38)	0.039
	Atherogenic index of plasma	0.03 ± 0.28	–0.03 ± 0.22	0.15 ± 0.34	<0.001

Normally distributed continuous variables are presented as means ± standard deviation, and continuous variables without a normal distribution are presented as medians (interquartile ranges). Categorical variables are presented as counts (percentages). NIHSS, National Institutes of Health Stroke Scale; HbA1c, glycated haemoglobin A1c; TC, total cholesterol; TG, triglyceride; HDL-C, high-density lipoprotein cholesterol; LDL-C, low-density lipoprotein cholesterol.

**Fig. 2.**
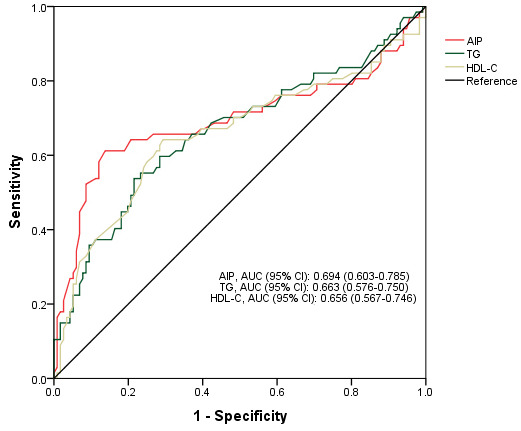
**Receiver operating characteristic curve of AIP for predicting 1-year functional outcome in patients with acute ischemic stroke receiving intravenous thrombolysis**.

**Fig. 3.**
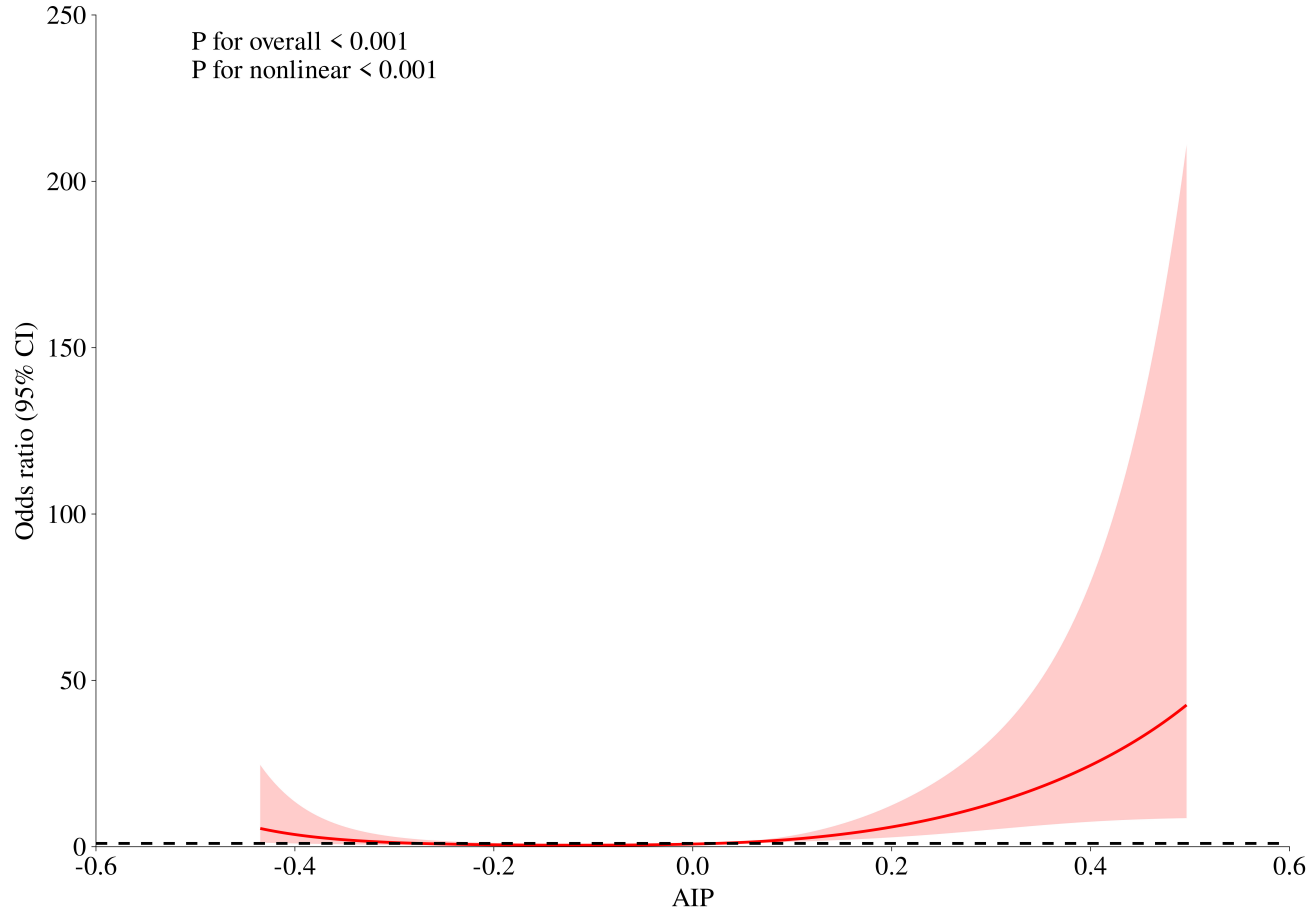
**Association between AIP and 1-year functional outcome in patients with acute ischemic stroke receiving intravenous thrombolysis using restricted cubic spline analysis**.

In the fully adjusted regression models, AIP as a continuous variable (odds ratio [OR]: 25.10; 95% confidence interval [CI]: 4.86–129.68) was associated with 1-year poor outcomes. When AIP was set as a categorical variable, ORs (95% CI) with the high AIP were 27.86 (9.33–83.25) for the prognosis compared with the low AIP (Table 2). ROC analyses showed that the best cut-off AIP value was 0.188. The sensitivity was 87.1%, the specificity was 61.2%, and the area under the ROC curve (AUC) of the AIP was 0.694 (0.603–0.785), which was preferable to other related lipid profiles (0.598 for TC, 0.663 for TG, 0.656 for HDL-C, and 0.592 for LDL-C) (Table 3 and Fig. 2). We also applied adjusted RCS plots to reveal the potential dose-manner associations between the AIP and poor 1-year outcome (Fig. 3). Here, we observed that high AIP was associated with a higher risk of 1-year outcomes, and a positive but non-linear trend was observed.

**Table 2.** **Association of AIP with 1-yaer functional outcomes in patients with acute ischemic stroke receiving intravenous thrombolysis**.

	Model 1		Model 2		Model 3	
	OR (95% CI)	*p *value	OR (95% CI)	*p* value	OR (95% CI)	*p* value
AIP	12.85 (3.70∼44.58)	<0.001	15.06 (3.90∼58.15)	<0.001	25.10 (4.86∼129.68)	<0.001
Low	1.00 (Reference)		1.00 (Reference)		1.00 (Reference)	
High	10.62 (5.11∼22.07)	<0.001	14.32 (6.23∼32.93)	<0.001	27.86 (9.33∼83.25)	<0.001

Multivariate logistic regression results are presented as ORs and 95% CIs. Model 1: Crude. Model 2: Adjusted for age, sex, BMI. Model 3: Adjusted for age, sex, BMI, and DBP, admission NIHSS, WBC, RBC, PLT, FPG, HbA1c, TC, LDL-C. OR, Odds Ratio; CI, Confidence Interval; AIP, atherogenic index of plasma.

**Table 3.** **Abilities of AIP, TC, TG, HDL-C and LDL-C for predicting 1-year functional outcomes in patients with acute ischemic stroke receiving intravenous thrombolysis**.

	AUC	95% CI	Sensitivity	Specificity	*p *value
AIP	0.694	(0.603–0.785)	0.871	0.612	<0.001
TC	0.598	(0.516–0.681)	0.250	0.955	0.053
TG	0.663	(0.576–0.750)	0.784	0.537	<0.001
HDL-C	0.656	(0.567–0.746)	0.707	0.642	0.008
LDL-C	0.592	(0.509–0.674)	0.293	0.925	0.065

Receiver operating characteristic curve results are presented as AUC with 95% CI, sensitivity, and specificity. AUC, Area Under the Curve; CI, Confidence Interval; AIP, atherogenic index of plasma.

## 4. Discussion

This is the first research focusing on AIP-related differences in the prognosis of AIS patients receiving IVT. High AIP was associated with poor 1-year functional outcomes. Additionally, we observed a positive but non-linear relationship between AIP and prognosis. These findings underscore the importance and need to consider the AIP levels when making medical decisions for AIS patients, consistent with the growing data suggesting that atherosclerosis plays a crucial role in AIS [16].

Atherosclerosis is the key cause of AIS and CVD [3, 4, 5, 6]. Thus, numerous lipid profiles have been used to evaluate the functional outcomes of AIS. However, traditional single index (TC, TG, HDL-C, LDL-C) as the evaluation of AIS was still limited and exhibited low predictive value. Small dense LDL-C (sdLDL-C) is significantly high associated with atherosclerosis, as is the case with AIS [17]. Furthermore, sdLDL-C detection is both difficult and costly, creating a need for an inexpensive and reliable tool to assess the degree of atherosclerosis in given patients. The AIP is a routine index that can indirectly reflect sdLDL-C levels [18]. Importantly, AIP can be easily computed using TG and HDL-C values, making it an inexpensive and reliable tool. The prognostic ability of the AIP has been suggested in CVD and the instability of carotid plaque [19, 20]. A 2024 study confirmed the associations of AIP with the 3-month outcomes of AIS [21]. However, no studies have examined the potential relationship between the AIP and long-term prognosis of AIS patients, regardless of receiving IVT. Our findings could extend the explanation, as we revealed that high AIP was linked to the 1-year poor outcome, with a positive but non-linear trend seen. When patients had high AIP, they had a higher risk of 1-year poor outcome compared with those with low AIP. Our study suggests that high AIP may offer greater predictive value than other related conventional lipid profiles. Given its ease of measurement and high predictive value, the AIP serves as an ideal tool for assessing patients with AIS, helping to better predict functional outcomes before IVT.

Despite the unclear mechanisms, several possible explanations may be proposed. High TG levels have been implicated in vascular subclinical atherosclerosis and might intensify the inflammatory reactions in both smooth muscle cells and vascular endothelial cells [22]. Conversely, HDL-C could exert multiple vasoprotective effects, including reducing apoptosis, mitigating inflammation, and protecting against oxidative stress [23]. Accordingly, the fact that AIP values offer simultaneous information obtained from patients’ TG and HDL-C levels could reflect pro-inflammatory and atherosclerotic effects modulated by high TG levels and the reduced anti-inflammatory HDL-C responses. Participants with high AIP tended to have higher BMI and HbA1c levels and were more likely to be smokers or drinkers, all of which led to AIS.

Our study, however, has several limitations. As a two-center, single-year investigation, its relatively small sample size may have limited the statistical power. Moreover, we did not assess the dynamic changes in AIP during hospital stay, an aspect that warrants further investigation in future studies.

## 5. Conclusions

In summary, high AIP was associated with poor 1-year functional outcomes in AIS patients receiving IVT, with a positive but non-linear relationship between AIP and prognosis. Future larger-scale studies are needed to clarify its clinical application.

## Availability of Data and Materials

The datasets are available from the corresponding author on reasonable request.
